# The use of the head louse as a remedy for jaundice in Spanish folk medicine: an overview

**DOI:** 10.1186/1746-4269-9-52

**Published:** 2013-07-22

**Authors:** José Ramón Vallejo, José Antonio González

**Affiliations:** 1 Departamento de Terapéutica Médico-Quirúrgica, Facultad de Medicina, Universidad de Extremadura, E-06006, Badajoz, Spain; 2 Grupo de Investigación de Recursos Etnobiológicos del Duero-Douro (GRIRED), Facultad de Biología, Universidad de Salamanca, E-37071, Salamanca, Spain

**Keywords:** Head louse, Jaundice, Ingestion, Traditional medicine, Spain

## Abstract

**Background:**

In Spain, head lice are considered a therapeutic resource for the treatment of jaundice. All folk remedies based on the ingestion of these insects meet in the present document, previously dispersed among a large number of references.

**Methods:**

An overview of the Spanish literature has been carried out. The most important databases have been consulted. All related works have been examined.

**Results:**

Although the method of preparation is diverse and the dose varies, the primary recommendation is a transference ritual consisting of taking nine live lice for nine days on an empty stomach without the patient’s knowledge. This traditional knowledge survives in Spanish society, and constitutes an example of the interrelation between Spanish and Latin American folk medicines.

**Conclusions:**

The survival of this therapy in the worldview of certain rural communities suggests the need to take into account the beliefs, ideas and behaviour patterns of popular culture in relation to health and disease.

## Abstract (es)

**Resumen (in Spanish):**

**Antecedentes**

En España, los piojos de la cabeza son considerados un recurso terapéutico para el tratamiento de la ictericia. Todos los remedios populares basados en la ingestión de estos insectos se reúnen en el presente documento, previamente dispersos por un gran número de referencias.

**Métodos**

Se ha llevado a cabo una revisión exhaustiva de la literatura española y han sido consultadas las bases de datos más importantes. Se han examinado todos los trabajos relacionados.

Aunque el método de preparación es diverso y la dosis varía, la recomendación principal es un ritual de transferencia consistente en tomar nueve piojos vivos durante nueve días en ayunas y sin el conocimiento del paciente. Este conocimiento tradicional sobrevive en la sociedad española, y constituye un ejemplo de la interrelación entre las medicinas populares española y latinoamericana.

**Conclusiones**

La supervivencia de esta terapia en la cosmovisión de algunas comunidades rurales sugiere la necesidad de tener en cuenta las creencias, ideas y patrones de comportamiento de la cultura popular en relación con la salud y la enfermedad.

## Background

Entomotherapy, understood as the use of insects or products derived from them for the treatment of human diseases [1], is currently one of the main focuses of ethnozoological research. Many authors have reported inventories of useful taxa, for example in Brazil more than 80 insect species are described [2], but they also document the importance of these animals in folk medicine, especially in rural areas. This is why research projects have been implemented in certain ethnic groups, human communities or cultures in which the use of medicinal insects and traditional knowledge about such species remain in force despite the risk of their attrition and risk of eventual disappearance [3,4].

Jaundice is not a disease but an external sign of an underlying pathological process that interferes with the normal functioning of the metabolism and excretion of bilirubin [5-7]. However, since antiquity it has been described as such–see Blánquez 1966 [8]–and folk medicine has treated jaundice using a wide variety of remedies from time immemorial. In Spain, according to George M. Foster [9], there are three main methods of healing jaundice: urinating on certain plant species, watching the water running on the bank of a river or stream, and drinking water containing human lice (*Pediculus humanus* Linnaeus, 1758). Among organisms of this taxon of blood-sucking insects (Phthiraptera: Anoplura: Pediculidae), two subspecies are differentiated: *P. humanus humanus* Linnaeus, 1758 (the body louse) and *P. humanus capitis* De Geer, 1778 (the head louse) [10,11]. Populations of these two lice can be separated based on a few morphological features, but mostly on the basis of certain behavioural and ecological differences. Head lice live and feed exclusively on the scalp, whereas body lice live on clothing and only move to the skin to feed [10,12,13]. This ecological differentiation arose when humans adopted frequent use of clothing [14]. In addition, molecular studies [15,16] confirm that head and body lice are conspecific, despite their differences. This point raises the first question: what subspecies is used as a medical remedy for jaundice? In 1929 Gárate [17] wrote: “… D. Miguel de Unamuno told me that several *Pediculi capitis* were poured into a glass, were crushed and water was added…” Subsequently, in 1972, Donostia clarifies that “live lice, taken from a clean and disease free head” are used [18]. These two direct references are sufficient to exclusively consider the use of head lice for the treatment of jaundice.

The life cycle of the head louse has three stages: egg, nymph, and adult. Adults are small (2.1–3.3 mm long), dorso-ventrally flattened and entirely wingless. Females can lay about 7 eggs per day, gluing nits to the base of human hair. To survive, adult lice need to feed on blood several times daily [10,12,13].

The objective of this paper is to collect in one document all traditional remedies used against jaundice based on the intake of adult head lice within the realm of Spanish folk medicine. These remedies, just as for other animal species, are scattered among countless references (many of which are inaccessible to the general public). It also analyzes the validity and geographical distribution of this therapeutic resource in Spain, and its relationship with the traditional medicine of other cultures.

## Methods

To get the maximum number of documentary sources, a qualitative systematic review of international and national databases was carried out. The international databases ISI Web of Science and Anthropology Plus were consulted. Domestic resources that were referenced include the database of Doctoral Theses TESEO, databases of the information system of Consejo Superior de Investigaciones Científicas (CSIC) and bibliographic web Dialnet. The overall search pattern covered the title, abstract and key words concerning ethnozoology related disciplines that have UNESCO codes (e.g., anthropology, history of medicine, zoology, entomology) and terms louse (lice), jaundice, folk medicine, folklore, digestology, ethnozoology, zootherapy and entomotherapy, in conjunction with the Spanish geographical scope. In turn, all related works systematically obtained were checked for recording data on the use of head lice as a remedy for jaundice.

## Results

A total of 33 studies were reviewed from the literature search. Table 1 provides a list of references consulted (arranged chronologically). The original descriptions of the remedies are included, and the geographical location of the use is indicated.

**Table 1:** List of Spanish traditional remedies for jaundice based on the ingestion of head lice

**References**	**Method of preparation and dosage**	**Geographical location**
Morán Bardón, 1927 [19]	9 lice are put into the patient’s lunch over the course of 9 days without his/her knowledge. They can be put into soup, an omelette or chocolate	Salamanca province
Gárate, 1929 [17]	Treatment consisting of ingesting lice (I was not told whether the lice were alive or not)	Bilbao (Biscay); Deva, Villabona (Guipúzcoa)
Risco and Rodríguez Martínez, 1933 [20]	Take lice. They forced the patient to take 9 lice	Melide (La Coruña)
Iribarren, 1943 [21]	Some healers prescribe taking a handful of lice or eating live lice with bread	Uitzi, Pamplona (Navarre)
Caro Baroja, 1944 [22]	An extremely disgusting procedure: take a variable number of lice	Vera de Bidasoa (Navarre)
Marcos de Sande, 1947 [23]	Ingest 7 live lice in a piece of bread	Garrovillas (Cáceres)
Díaz Mora, 1948 [24]	The patient was administered 3 crushed lice dissolved in “aguardiente” (liqueur) or urine. In Gata the number of parasites ingested for correct treatment is 16	Valverde del Fresno, Eljas, Gata (Cáceres)
Lis Quibén, 1949 [25]	Ingest lice. In Cotovad, for 9 days, they give the patient on an empty stomach a glass of wine with 9 lice that were left outside in the cold the night before. In Cambados, the patient eats dead lice in a broth or alive in bread. In Verín, they give the patient the noted 9 lice in milk or chocolate	Verín (Orense); Cotovad, Cambados (Pontevedra)
Arias, 1955 [26]	Take 9 lice for 9 days	Cornellana (Salas, Asturias)
Duran Sotelo, 1961 [27]	Take lice	Santa Justa–Moraña (Pontevedra)
Taboada, 1961 [28]	It is recommended to take 9 lice mixed in milk or chocolate	Verín (Orense)
Fernández-Ruiz, 1965 [29]	Medication consisting of taking lice with chocolate	Asturias
Donostia, 1972 [18]	Put 5 live lice, taken from a clean and disease free head, in the heart of an apple or in a piece of bread and eat them. If 5 is not enough, the next time 7 lice are used	Amaiur (Navarre)
Barandiarán, 1974 [30]	They say that to cure jaundice it is necessary to ingest lice	Basque Country
Barriola, 1979 [31]	Lice should be administered without patient knowledge, in chocolate, wine or liquor, soup, or a corn silk infusion	Basque Country
Becoña Iglesias, 1981 [32]	The patient should take lice without his/her knowledge. The quantity to be taken was 3 or 4 lice, once or twice. They were given in water or milk	A Comboa, Estribela, Rorís (Pontevedra)
Carril, 1981 [33]	They gave the affected person without his/her knowledge, 9 lice for 9 consecutive days at breakfast or lunch	Salamanca province
Goicoetxea Marcaida, 1983 [34]	The patient should ingest lice, better living, taking them with liquor, chocolate or the decoction of corn silk	Amorebieta, Elorrio, Zeanuri (Biscay); Olaeta (Álava); Irún (Guipúzcoa)
Blanco, 1985 [35]	Pour lice into broth; eat lice	El Arco, Negrilla de Palencia, Robleda (Salamanca)
Junceda Avello, 1987 [36]	A disgusting recipe: take 9 lice for 9 days. Eat 9 lice together with vegetables	Asturias; Galicia
Martí i Pérez, 1988 [37]	Ingest live lice mixed with some food	Dosrius, Barcelona city (Barcelona); Blanes (Gerona)
Vázquez Gallego, 1989 [38]	While fasting, give the patient over the course of 9 days, a glass of wine with 9 lice that were left outside in the cold the night before	Galicia
Barandiarán, 1990 [39]	It is necessary to eat lice. The patient should drink water in which 7 head lice were previously cooked	Basque Country
Mateos Romero, 1990 [40]	For 3 days, eat soup prepared with oil previously used to fry 8 or 10 lice. The patient should not know the origin of the recipe	Torremenga (Cáceres)
Rúa Aller and Rubio Gago, 1990 [41]	Drink water in which lice had been cooked	Comarca de El Bierzo (León)
Carril, 1991 [42]	Drink a lice decoction	Fuenteguinaldo (Salamanca)
Guío Cerezo, 1992 [43]	Put lice into a fig and eat it	Llerena (Badajoz)
Fresquet, 1995 [44]	Some people gave children a drink made of white wine with lice, cinnamon and sugar	Comarca de la Ribera Alta (Valencia)
Pámpano and Redondo, 1997 [45]	9 live lice are eaten mixed with flour, honey and sugar. Afterwards, a diet of varied stocks should be followed	San Vicente de Alcántara (Badajoz)
Erkoreka, 2002 [46]	Live lice should be added to porridge, chocolate, coffee or other food to be taken by the patient without his/her knowledge	Basque Country
Álvarez Peña, 2004 [47]	Swallow live lice with milk or butter	Deva (Gijón) and other councils from Asturias
Barandiarán and Manterola, 2004 [48]	In Amorebieta, Bera and Elorrio, in the first decades of the 20th century, it was necessary to give the patient several lice in a small cup of chocolate or mixed with liquor without his/her knowledge: 7, 9, 11, 13, always an odd number. The same remedy was applied in Zeanuri and Olaeta, where lice were introduced into soup, and Quintana where they were placed in the patient’s ordinary food without his/her knowledge. In villages far from the coast for several days, the patient was given a tisane in which 9 lice were placed or they were mixed with some food eaten regularly. Our current surveys have also collected this remedy. In Bermeo and Hondarribia live lice were added to porridge, chocolate, coffee or other food that should be eaten by the patient without his/her knowledge. In Orozco the lice were eaten in an omelette	Amorebieta, Bermeo, Elorrio, Orozco, Zeanuri (Biscay); Olaeta, Quintana (Álava); Hondarribia (Guipúzcoa); Bera (Navarre)
Álvarez Caperochipi, 2012 [49]	At the beginning of the XX century, many healers applied dried head lice dissolved in water. They crushed the lice in a glass and then added water. They would give a drop to the patient and if he/she felt good they would give the patient two drops the following day. The dosage would be increased daily by one drop each day up to 21 drops to then be decreased	Ilarregui and other villages from Navarre

### Validity

The therapeutic use of head lice against jaundice has remained in Spanish oral tradition and has survived until today, although its current use has not been documented in research within the last decade.

### Method of preparation and dosage

In 9 studies (27%) the necessary intake of live lice is documented. It is a transference therapy based on the belief that these insects feed on bile, which is the materialization of the “disease” [18,25,50].

Many references indicate that lice should be administered to the patient on an empty stomach without his/her knowledge. They are usually dissolved or mixed in food that the patient has to take. A wide range of food is mentioned and chocolate is especially recommended.

With regard to the dosage, 16 studies (48%) indicate the number of lice to ingest. In most cases, odd numbers are mentioned; in nine the recommended number is 9. This is the same amount that appears most often as the recommended number of days for treatment duration (9 days). In Spanish popular culture, the odd are laden with symbolism and considered to be sacred in character, thus, they form part of healing rituals. The number 9 is considered as “the perfection” and a symbol of life [51].

### Geographical distribution

**Figure 1:**
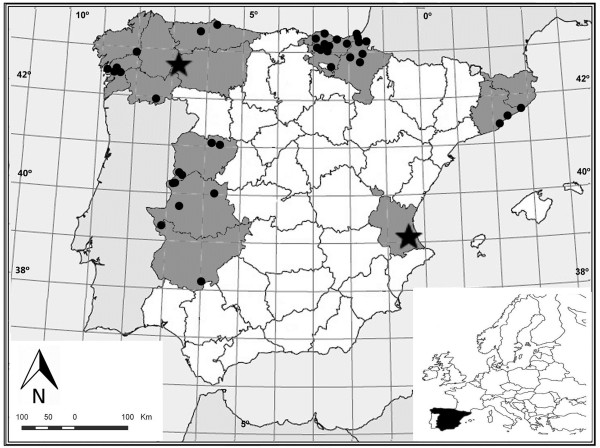
**Geographical distribution of the use of head lice as an anti-jaundice remedy in Spain.** The cited provinces and autonomous communities are highlighted in gray; (**●**) mentioned localities; (**★**) “comarcas” (~ regions, shires).

As for the geographical distribution of the use of head lice as a remedy for jaundice, appointments found cover much of the northwestern quadrant of Spanish territory (Figure 1). Numerous localities in the Basque Country and Navarre are mentioned.

Numerous authors show that using animal resources as therapy is a widely distributed atavistic practice [52,53], a fact that is demonstrated by the use of head louse against jaundice in distant geographical areas [54,55]. Its use is particularly common in the Hebrew culture. Izaak Walton in his famous book “The Compleat Angler” (published in 1653) records that the Jews were the first to say that swallowing live lice is a good remedy for jaundice [56]. For their part, the German rabbi Yair Bacharach (1639–1702), author of the collection of responsa known as “Havvot Yair” (“Villages of Yair”), indicates that the patient should take 8 lice taken from his/her own head–see Rosner and Bleich 2000 [57]. Ben-Ezra in 1949 recorded in Horodetz (one of the oldest Jewish communities in Russia-Poland) the introduction of lice in an omelette as treatment [58]. In Latin America there are also references to this medical practice. In this case, the remedy would have been brought by the Spanish conquistadors and assimilated by the Spanish American folk medicine in an eclectic form [9]. The recommended number of specimen to take in Chile, Peru, Guatemala and Argentina is 4 or 5 with examples found using measures such as a thimbleful [59-62].

## Discussion

The key elements of Spanish folk medicine can be found in beliefs and empiricism [50,63], thus the employed remedies and recipes have been developed primarily through magical conceptions using the homeopathic principle *similia similibus curantur* or following the natural-philosophical basis of the humoral theory [64]. Keep this in mind, and although the explanations and justifications for taking head lice as an anti-jaundice remedy are complex, it can be hypothesized following two arguments. First, an Aristotelian scheme of thought like Bartholomaeus Anglicus (born before 1203–died 1272) can be used as a starting point. Anglicus applied the humoral theory to the origin of human louse and proposed a hypothesis with a magical explanation of homeopathic type. In chapter LXXXII of his compendium *De proprietatibus rerum* (“On the Properties of Things”), this author wrote: “lice are begotten of rotten humours that are between the skin and flesh, and fall outside with the sweat”–see Seymur 1975–1988 [65]. Furthermore he classifies them, stating that “those who are begotten from the choleric humour are greenish-yellow”. Choleric humour is synonymous of yellow bile, hence following the principle *similia similibus curantur* these ectoparasitic insects can be considered a remedy for the treatment of jaundice. This type of reasoning, based on a magical explanation (without rational basis), has been important in therapy throughout history and was highly valued by Classical Greek philosophers such as Empedocles or Plato [66,67]. Second, folk medicine users also create their own conceptions about health by developing theories with rational bases. For example, in the early 20th century in the Basque Country people still thought that a web in the liver or stomach prevented the normal circulation of bile making it necessary to drill or destroy it. For this reason, the basis of curative applications lies in the belief that the patient should ingest live lice to either break the web or eat it [34,48]. In Navarre it was explained that when the lice reached the initial part of the intestine they would cause a reaction and help release retained bile [49]. These popular explanations coincide with the description given by Sterpellone [68], who presumed that: “… swallowed lice reaches the stomach and, as they are resistant to the action of gastric juices, pass through the pylorus and enter the duodenum, tickling the walls. This tickle causes contractions in the duodenal wall favouring bile flux and relieving bile stasis which causes jaundice when the bile ducts are blocked due to a mucous stopper.”

In Early Modern history lice intake was described as a “superstitious remedy”, i.e. as a misconception and not accepted by official medicine, which considered it an “illegitimate knowledge”. Despite all, this remedy, typical of oral tradition, has been passed on to us (see Table 1).

At present, folk medicine makes use of the Information and Communication Technology for the transmission and dissemination of knowledge. It is interesting to note that inquiries related to this folk remedy appeared on various websites relating to health and disease management (http://www.home-remedies-for-you.com, http://www.medicalfaq.net) in June 2008. The question posed by a user was as follows: “Can head lice given to a person reduce extreme jaundice? Is it true? Please, advise me.” As expert, G M (on June 12, 2008) responded: “I have never heard of any such treatment for jaundice involving head lice. Preferring ‘natural’ cures has its place, but everything that is natural is not necessarily good” (see http://www.home-remedies-for-you.com/askquestion/18048/jaundice-treatment-and-diet-help-can-head-lice-giv.html). The previous question and answer reflect popular conceptions about health and disease. Considering that this remedy has been part of our collective knowledge and cultural background, we can state that it is part of a deeply rooted “implicit theory”. Therefore, although the remedy is not currently used in Spain, it is necessary to consider how these ideas have influenced the behaviour of the patients, how they cope with the disease and how to address medical pluralism.

Moreover, this work lies within the context of ethnomedicine and insects, the latter being an animal group of great therapeutic potential. In recent years there have been regularly published reports addressing their importance in the search for new drugs [69-71] and even the pharmacological properties of certain species [72-74] or insect-derived products [75,76] have been studied. Thus, the present document should serve as a basis and a valuable tool for future chemical screening and biological assays, and should open new perspectives for the economic and cultural assessment of animals traditionally thought of as being of no use.

## Conclusions

The use of folk remedy for jaundice based on the ingestion of head lice is difficult to understand due to lack of information on sympathetic or antipathetic associations that were established in the past. However, the survival of this traditional knowledge in Spanish society and its relatively wide geographical distribution helps verify the consistency of the theories formulated within rural communities, even though they may conflict with biomedical health care. It also suggests the need of taking into account the beliefs, ideas and behaviour patterns of popular culture in relation to health and disease for a better understanding with the patient.

## Competing interests

Both authors declare that they have no competing interests.

## Authors’ contributions

The two authors contributed equally during the data collection, data analysis and preparation of the manuscript, and read and approved the final manuscript.
